# A pandemic risk index to improve supply chains decision-making between US and Mexico: A COVID-19 case study

**DOI:** 10.1371/journal.pone.0327526

**Published:** 2025-09-11

**Authors:** Billy Hernawan, Guillermo F. Duran-Sierra, Enrique Zarate-Losoya, Zenon Medina-Cetina, Miriam Olivares, Maria J. Perez-Patron, Matt Cochran, Gregory Pompelli

**Affiliations:** 1 Zachry Department of Civil & Environmental Engineering, Texas A&M University, College Station, Texas, United States of America; 2 Harold Vance Department of Petroleum Engineering, Texas A&M University, College Station, Texas, United States of America; 3 Yale Library, Yale University, New Haven, Connecticut, United States of America; 4 Center for Economic Studies (CES), U.S. Census Bureau, Washington, District of Columbia, United States of America; 5 Cross-Border Threat Screening and Supply Chain Defense, Texas A&M University, College Station, Texas, United States of America; Villanova University, UNITED STATES OF AMERICA

## Abstract

The Social Vulnerability Index (SVI) developed by the Centers for Disease Control and Prevention (CDC), has been widely used as a benchmark to measure the state of vulnerability of counties across the United States. The SVI is integrated using a simple aggregation methodology on a set of variables reflecting the region’s socioeconomic status, household characteristics, racial & ethnic minority status, and housing type/transportation. Due to its simple construction and inclusion of significant variables publicly available, the SVI has grown exponentially in popularity among organizations and government officials as a tool for decision-making, especially for resource allocation and for regional risk assessment. Furthermore, the COVID-19 pandemic brought a set of unprecedented challenges in the bi-national health between the United States and Mexico, particularly on the state of risk of supply chains. Since the North American Free Trade Agreement (NAFTA) became effective in 1994 and then renewed in 2020 as USMCA, Mexico has grown to be the biggest trading partner of the U.S., fast approaching a trade value of more than a trillion USD a year. For which conducting regional risk assessment following the SVI formulation can be a significant impact for multiple stakeholders and organizations. In this work, the formulation of the SVI is analyzed using a risk framework as a reference, to corroborate its applicability for decision-making, and to expand it to account for variables and processes impacting supply chains during the COVID-19 pandemic. This analysis shows that vulnerability is only one of three factors required to conduct risk assessment (i.e., hazards vulnerability, and consequences), needed to produce a baseline of reference to make informed decisions. A case study is also developed based on the use of the SVI during the COVID-19 pandemic for supply chains between the U.S. and Mexico, by introducing the formulation of a risk index that is compatible with the proposed risk framework. The first step to expand the SVI into a risk index for supply chains between U.S. and Mexico, was to reproduce the CDC methodology, followed by using an Empirical Cumulative Density Function (ECDF) aggregation methodology to justify it statistically, and then to illustrate its benefits and limitations when extended into a new risk index (accounting for the three required risk components). As a result, a bi-national risk index map is produced after harmonizing publicly available variables in the U.S. and Mexico, illustrating the potential to quantify the state of regional risk for supply chains and other path-dependent systems, and setting a reference to further improve it.

## Introduction

Measurement and quantification of vulnerability of regional economic, social, and environmental systems, is of significant interest for the public and private sectors. This is often a challenging task due to their dynamic, complex, and inter-dependent nature, which is conditioned on specific threats, either of natural or anthropogenic origin, and specific metrics of impact. One possible approach in quantifying the vulnerability of a given system is through the aggregation of relevant variables into a single index that gauges the level of vulnerability of the themes they represent, making possible to map the resulting regional aggregation (although not formally accounting for their inter-dependency). The proposed index variables usually fit relevant subcomponents or subthemes of vulnerable systems, such as socioeconomic status, state of infrastructure, and population demographics. That is the case of the Social Vulnerability Index (SVI), developed by the Centers for Disease Control and Prevention (CDC), which was formulated based on the percentile ranking method [1]. This has been extended to include other systems to measure wildfire vulnerability, natural disaster vulnerability, and obesity prevalence, among others [2–4]. However, the SVI has showed some limitations, as was the case, when validated using outcomes from Hurricane Sandy [5]. Alternatively, some researchers have developed other types of vulnerability indexes using, for instance, Principal Component Analysis. This is the case of the Social Vulnerability Index (SoVI) [6], which was constructed by reducing the dimensions of the input data to assess environmental threats, and which has been widely adopted to investigate various other threats [7,8]. Both indexes are valuable mainly due to their simple formulation and because of using publicly available datasets. Others have taken a similar concept of aggregation to formulated similar vulnerability indexes for different applications [9–11].

A system risk interpretation of vulnerability is defined as a function of three components needed to reproduce decision-making: *Risk (Hazard, Vulnerability, Consequences)* [12]. Where *Hazard* is the probability of reaching a given intensity of a driving change event or Threat, *Vulnerability* is the probability of reaching a System damage level (i.e., social, economic, and/or environmental) conditioned on the occurrence of a given Threat intensity, and *Consequences* is the Metric of Impact value associated to repairing the system under consideration, or its loss when subjected to a given System’s *Vulnerability* or damage level. That is, a baseline of reference of a System or estimate of a System state of risk is needed to fully reproduce decision-making, and as such requires three components to define *Risk* (*Hazard, Vulnerability, Consequences*) [12].

Due to the unprecedented spread of the COVID-19 pandemic that affected global supply chains [13], there was a pressing need for conducting regional risk assessment using a proven framework, and available risk components to estimate supply chain risk scenarios, particularly, between the U.S. & Mexico border where it saw an initial 88% contraction in the imports from the U.S. to Mexico due to manufacturing disruption [14], border restrictions and logistics [15]. This work presents a discussion to analyze the potential of the SVI as a simple and reliable tool to use it as a basis to formulate preliminary regional risk assessment.

Moreover, supply chain networks in global trade have led to critical interdependencies between nations largely due to the rise of globalization [16]. Consequently, a supply chain network links several vendors and clients, and any major disruptions in any part of the supply chain System often lead to severe economic and social losses [17]. Efforts have been made to analyze supply chain systems using the social vulnerability index, specific to a given region of interest [18]. Therefore, a development of a COVID-19 pandemic risk index is critical in helping in decision-making with regards to supply chain logistical planning, and reinforcing the resilience of the binational supply chain.

To the best of the authors’ knowledge, there is no prior work conducted analyzing the statistical properties of the CDC’s SVI index. The overarching objective of this paper is to formulate a risk index inspired in the concept of the SVI, and compatible with a risk framework [19]. The first section of this paper explores the reproducibility of the SVI using publicly available data, and looks at SVI’s statistical properties as an aggregated index to justify its extension into a risk index. The subsequent section introduces the formulation of a risk index based on the SVI aggregation method, by incorporating a risk framework and by adding variables that expand the original SVI methodology to account for the COVID-19 Threats and impacted public health Systems [20]. This new *Risk* index accounts for the three risk factors needed to reproduce decision-making: Threats → *Hazard*, Systems → *Vulnerability*, and Metrics of Impact → *Consequences*. And is applicable for both the U.S. and Mexico. Finally, synthetic examples demonstrate a path-dependent regional risk assessment to illustrate the applicability of the proposed risk index to supply chain Systems. The paper ends with a discussion of the major findings, differences between the proposed risk new index and the SVI, and the advantages and limitations of their applicability.

## Methodology

### Data sources

This study is based on various datasets that have been collected based on community surveys. All of the data that were used in the SVI construction were retrieved from the 2014−2018 American Community Survey (ACS) 5-year estimate. Relevant datasets were streamed through the census API filtered by variable ID. Epidemiological datasets that include disease prevalence and healthcare capacity indicators were collected from various governmental agencies such as the CDC’s Behavioral Risk Factor Surveillance System (BRFSS), and from the Health Resources and Services Administration (HRSA). Most of the data that was collected at the county level occurs annually. Lastly, during the COVID-19 pandemic, the CDC released 7-day estimate of COVID-19 cases per 100,000 daily, and for this study the data from 4^th^ of August 2021 was included in the index creation to illustrate its applicability. All of the dataset was then compiled into one dataset, and that counties with missing data were excluded (mainly counties in Puerto Rico).

### Percentile ranking method

The CDC methodology to formulate the SVI involves the construction of the following subthemes indexes: socioeconomic status index, household composition/disability index, racial & minority status/language index, and housing/transportation index. The SVI estimation process begins by converting a total of 15 variables (maps) to percentile ranks using values from all the U.S. counties. Then these are scaled down to a range between 0–1 using a uniform distribution. These scaled values are assigned back to their respective counties and summed according to each of the four subtheme groups. The SVI assumes equal contribution from all 15 variables without assigning weights to any of them [1]. The equation to convert each variable to a percentile rank is given in Equation 1 below,


$$Percentile\;Rank=\frac{Rank-1}{N-1}$$ (1)

where N is the total number of data points. The final SVI is constructed by adding the sums of all subthemes and converting the result into a new percentile rank using data from all counties. The corresponding ranking is then assigned back to each county, resulting in the SVI map. From the construction process point of view of the SVI, it is unquestionable that its major advantage for using it is its very simple computational implementation compared to other risk index construction methods.

### SVI construction using the Empirical Cumulative Density Function (ECDF)

**Fig 1:**
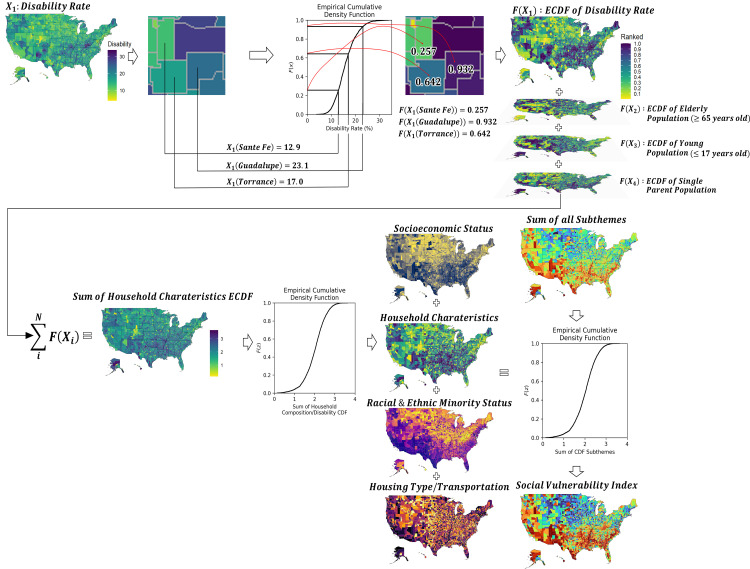
Workflow of SVI Construction using ECDF. Variables were initially transformed to a scale from 0 to 1 using ECDF. Maps of socioeconomic status, racial & ethnic minority status and housing type/transportation were also constructed using variables classified under their respective subthemes. The SVI is then computed using the ECDF-scaled summation of all four subthemes.

To explore the statistical meaning of the SVI index formulation, it is proposed to use the Empirical Cumulative Density Function (ECDF) for each SVI participating variable assigned to each SVI’s subtheme. The ECDF can be statistically interpreted as an empirical function representing the population of a given variable (in this case, the population size is 3,142 counties). Then, at the county level, the corresponding variable’s ECDF cumulative probability value (i.e., its cumulative relative frequency) is assigned back to each county to produce the variable’s frequency or ‘probability map.’ Next, all frequencies or probabilities assigned to each variable within a subtheme are summed, and the corresponding ECDF is computed. Similar to the process done at the variable level, the probabilities produced from each subtheme ECDF are read and mapped back to its corresponding counties. Finally, all ‘probability maps’ of each subtheme are summed up, and the ECFD of the corresponding sum is produced. Using each county’s sum value, its corresponding frequency or probability value is read and mapped back to the county, producing the same SVI map as the one produced using the CDC’ ranking method. The workflow of this estimation method is illustrated in Fig 1, where the construction of one of the subthemes (i.e., household characteristics) is used as an example to show in detail the aggregating process used for the other subthemes, to finally produce the SVI.

By following the ECDF approach to compute the SVI, a formal statistical treatment to the SVI participating variables is given, allowing for the visualization of the shape of the population of each variable, and opening an opportunity to model it using suitable probability density functions (e.g., Gaussian, Lognormal, etc.). Moreover, by using probabilities to represent events of a given population, it is then possible to operate across variables to define new populations using basic probability theory. At the same time, it makes evident the assumptions made in the construction of the SVI, for instance, the aggregation process of variables within a subtheme creates a new variable (i.e., the sum of the ECDF probabilities of each variable), which is subjected to probability theory of sums of variables or events, as they change in space (and time). Also, it explicitly shows the independence assumption made for all participating variables (i.e., no cause effect analysis is considered).

### Classification of variables to formulate a risk index

Table 1 lists the 32 variables proposed to define a pandemic social risk index for supply chains in the US. This is inspired by the SVI aggregation methodology, and includes all the 15 CDC’s SVI variables (marked with an asterisk), showing their corresponding subthemes. The complementary 17 variables are intended to extend the scope of the SVI to account for the social state of risk during a pandemic like COVID-19. Also, each variable in the table is classified according to the risk framework introduced in the previous section. The goal is to create a set of three new themes following the classification of the *Risk* factors: Threats → *Hazard*, Systems → *Vulnerability*, and Metrics of Impact → *Consequences*.

**Table 1:** Variables considered in the formulation of the risk index (* indicates metrics included in the formulation of the SVI).

#	Variables	Category	Data Source	SVI Subtheme
1	Persons below poverty *	Threat	ACS Est-5 2014–2018	Socioeconomic Status
2	Civilian unemployment *	Threat	ACS Est-5 2014–2018	Socioeconomic Status
3	Annual Per capita income *	Threat	ACS Est-5 2014–2018	Socioeconomic Status
4	Persons with no high school diploma *	Threat	ACS Est-5 2014–2018	Socioeconomic Status
5	Persons aged 65 and older *	System	ACS Est-5 2014–2018	Household Composition/Disability
6	Persons aged 17 and younger *	System	ACS Est-5 2014–2018	Household Composition/Disability
7	Civilian noninstitutionalized population with a disability *	System	ACS Est-5 2014–2018	Household Composition/Disability
8	Single parent household with children under 18 *	System	ACS Est-5 2014–2018	Household Composition/Disability
9	Minority (all persons except white, non-Hispanic) *	System	ACS Est-5 2014–2018	Minority Status/Language
10	Persons (age 5+) who speak English “less than well” *	System	ACS Est-5 2014–2018	Minority Status/Language
11	Housing in structures with 10 or more units *	System	ACS Est-5 2014–2018	Housing/Transportation
12	Mobile homes estimate*	System	ACS Est-5 2014–2018	Housing/Transportation
13	At household level (occupied housing units), more people than rooms *	System	ACS Est-5 2014–2018	Housing/Transportation
14	Households with no vehicle available *	System	ACS Est-5 2014–2018	Housing/Transportation
15	Persons in institutionalized group quarters *	System	ACS Est-5 2014–2018	Housing/Transportation
16	Estimated number of people per square mile	Threat	ACS Est-5 2014–2018	–
17	COVID-19 Cases per 100k - last 7 days	Threat	COVID-19 Community Profile Report 2020–2021	–
18	Number of households	System	ACS Est-5 2014–2018	–
19	Persons who are uninsured	System	ACS Est-5 2014–2018	–
20	Persons employed in healthcare support occupations	System	ACS Est-5 2014–2018	–
21	Persons ever diagnosed with Chronic Obstructive Pulmonary Disease	System	CDC BRFSS 2018	–
22	Persons ever diagnosed with diabetes	System	CDC BRFSS 2018	–
23	Persons ever diagnosed with high blood pressure (hypertension)	System	CDC BRFSS 2017	–
24	Persons reporting to be obese (BMI > 30)	System	CDC BRFSS 2018	–
25	Persons reporting to have asthma	System	CDC BRFSS 2018	–
26	Rate of primary care physicians per 1,000 people	System	CDC BRFSS 2018	–
27	Rate of advanced practice registered nurses per 1,000 people	System	HRSA 2019	–
28	Rate of hospital beds per 1,000 people	System	HRSA 2019	–
29	Number of Federally Qualified Health Centers per 1,000 people	System	HRSA 2019	–
30	Persons ever diagnosed with chronic kidney disease	System	CMS 2018	–
31	Number of intensive care unit (ICU) beds per 1,000 people	System	Kaiser Health News 2019	–
32	Population estimate	Metric of Impact	ACS Est-5 2014–2018	–

All data supporting these variables was acquired from the U.S. Census Bureau, as it was available during the COVID-19 pandemic. To validate the CDC methodology to assess the SVI, a first step consisted in reproducing its computation using the R programming language (i.e., the 2018 edition of the CDC’s SVI).

The classification of the SVI variables according to the risk framework, showed that 4 variables classified as Threats and 11 classified as Vulnerable Systems (11). There were no variables classified as Metrics of Impact. Notice that from a decision-making point of view, a risk classification validates the definition of a state of risk (i.e., needs the participation of the three risk components), and helps to best interpret the scope and applicability of the resulting index. This analysis shows that the SVI is a combination of variables representing drivers of change or Threats and Vulnerable Systems, which supports both the *Hazard* and *Vulnerability* assessments, respectively. Therefore, the *Consequences* component is missing in the SVI. This confirms that the definition of the CDC’s SVI agrees with the definition of *Vulnerability* from a risk and decision-making perspective.

After the classification of the SVI variables is conducted according to the risk framework, it is proposed to introduce additional variables i) to extend the SVI original scope to account for the effects of the COVID-19 pandemic, and ii) to account for the Consequences risk component to extend its scope to a Risk index. For this latter, and to explore the applicability of the risk index, it was proposed to use the ‘population estimate’ variable as a social Metric of Impact, to then produce a social state of risk associated with the COVID-19, to allow for the regional estimation of the state of risk of a supply chain in terms of impacted population.

The criteria to select the additional variables that extends the CDC’s SVI formulation to account for COVID-19 as an epidemiological Threat and for the vulnerability of the healthcare *System*, was based on the work by Acharya & Porwal [21]. Table 1 includes 17 additional variables representing these components. Some epidemiological variables such as the prevalence of certain diseases and conditions were considered contributing factors to the population’s vulnerability to COVID-19 [22–27]. Notice that COVID-19 spatial data were retrieved from the COVID-19 Community Profile Report [28]. Variables addressing the vulnerability of the healthcare System were included as well [29,30], reflecting the role of the COVID-19 Threat as a stressor to handle a significant and sudden influx of patients (i.e., hospitals and healthcare personnel), and consequently reflecting the state of vulnerability of the healthcare System. Finally, the population estimate was considered a Metric of Impact to add the *Consequence* assessment consistent with the proposed risk framework [31,32].

Once all variables of all three risk subthemes were defined, then it was possible to estimate the pandemic social risk index using the same aggregation technique as the SVI, but based on the ECDF formulation approach instead. The same procedure was also applied independently to Mexico, albeit with a smaller number of variables due to the inherent difference in the availability and risk classification of variables. Such differences led to an additional step in the process of creating a COVID-19 pandemic social risk index map between the U.S. and Mexico, defined as a binational variable harmonization process, discussed in the following section. The goal was to ‘remove the border’ between the U.S. and Mexico, to map a single region using the same risk index, to be able to compare different COVID-19 health regional states of risk.

### Harmonization of risk variables between US and Mexico

In order to produce a continuous risk index map between the U.S. and Mexico, it was needed to ‘harmonize’ the variables defining their corresponding COVID-19 social risk: Threats, Systems, and Metrics of Impact, between U.S. and Mexico. Previous efforts have formulated a social vulnerability index for Mexico. Sierra-Alcocer et al. [33], using the partial least square technique, aggregated temporal variables that were subdivided into 4 subthemes: socioeconomic, housing & hygiene, health care, and epidemiological. Recently, León-Cruz et al. [34] used principal component analysis and a total of 18 variables to produce a spatial and temporal social vulnerability index to natural Threats. This work adopted a subset of the variables in Mexico that can be matched or ‘harmonized’ with the variables that were used to construct the U.S. pandemic social risk index described in the previous section. Due to the differences in demographics, healthcare and social welfare Systems, it was noted that some variables were similar but not identical in meaning. For example, “Persons older than 5 years old who speak English less than well in the US” and “Persons older than 3 years old who speak Spanish less than well in Mexico”, addressed a similar characteristic of the population in the U.S. and Mexico respectively, but had not the same meaning. Notice that most of the SVI variables contain the same type of information for both the US and Mexico. Table 2 lists the resulting 18 harmonized variables that were used in the index aggregation process to produce the bi-national health regional risk index due COVID-19. All 18 variables listed in Table 2 are a subset of the variables that were used to construct the risk index described above and were listed in Table 1.

**Table 2:** Harmonized metrics of the US and Mexico.

#	Metrics	US Data Source	Mexico Data Source	Risk Classification
1	Persons below poverty	ACS Est-5 2014–2018	Coneval 2015	Threat
2	Civilian unemployment	ACS Est-5 2014–2018	INEGI Census 2020	Threat
3	Persons with no high school diploma	ACS Est-5 2014–2018	INEGI Census 2020	Threat
4	Estimated number of people per square mile	ACS Est-5 2014–2018	INEGI Census 2020	Threat
5	COVID-19 Cases	COVID-19 Community Profile Report 2020–2021	Datos Abiertos	Threat
6	Persons aged 65 and older	ACS Est-5 2014–2018	INEGI Census 2020	System
7	Persons aged 17 and younger	ACS Est-5 2014–2018	INEGI Census 2020	System
8	Civilian noninstitutionalized population with a disability	ACS Est-5 2014–2018	INEGI Census 2020	System
9	Persons (age 5+in US or 3+ in Mexico) who speak English in US/Spanish in Mexico “less than well”	ACS Est-5 2014–2018	INEGI Census 2020	System
10	Overcrowding rate	ACS Est-5 2014–2018	INEGI Census 2020	System
11	Population estimate	ACS Est-5 2014–2018	INEGI Census 2020	Metric of Impact
12	Number of households	ACS Est-5 2014–2018	INEGI Census 2020	System
13	Persons ever diagnosed with Chronic Obstructive Pulmonary Disease (Only among COVID-19 patients in Mexico)	CDC BRFSS 2018	Datos Abiertos	System
14	Persons ever diagnosed with diabetes (Only among COVID-19 patients in Mexico)	CDC BRFSS 2018	Datos Abiertos	System
15	Persons ever diagnosed with high blood pressure (hypertension) (Only among COVID-19 patients in Mexico)	CDC BRFSS 2017	Datos Abiertos	System
16	Persons reporting to be obese (BMI > 30) (Only among COVID-19 patients in Mexico)	CDC BRFSS 2018	Datos Abiertos	System
17	Persons reporting to have asthma (Only among COVID-19 patients in Mexico)	CDC BRFSS 2018	Datos Abiertos	System
18	Persons ever diagnosed with chronic kidney disease (Only among COVID-19 patients in Mexico)	CMS 2018	Datos Abiertos	System

### Path-dependent supply chain risk assessment

Once a pandemic social risk index was available that depicted the three components needed to assess a reference for decision-making, it was then possible to use the resulting risk index map to produce specific estimates of the impacts of other systems overlaying it. This can be done for a given path between any two geographical locations defined between origin and destination. Transportation networks, which are Vulnerable Systems for supply chains while subjected to different Threats, can be strained and damaged which could produce significant Impacts or losses [35,36].

**Fig 2:**
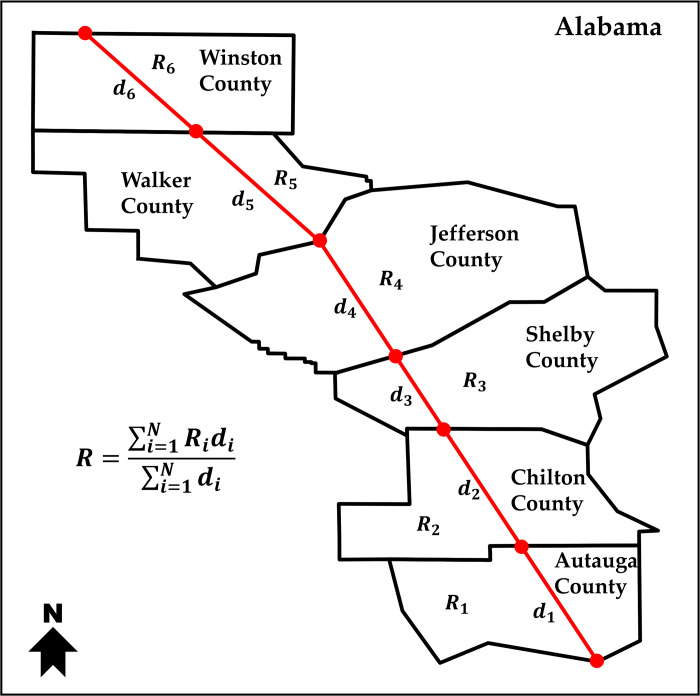
Estimation of a path-dependent supply chain state of risk. The path-dependent supply chain state of risk is a function of the distance traveled across each county and the pandemic social risk index associated with each county.

As a preliminary practical application of the proposed pandemic social risk index map, a path-dependent risk assessment was conducted in a supply chain. The risk along the path connecting the destination of a supply chain is calculated by taking the summation of the risk index county values multiplied by the distance of the path crossing the county, for all counties crossed by the path. This value was then normalized by the total distance between the origin and destination (i.e., weighted average by the total distance). Fig 2 highlights the workflow of this weighted risk assessment computation. To illustrate the applicability of the pandemic social risk index, two synthetic examples are presented that show the estimation of a path-dependent health regional risk for one possible path from Saltillo, Coahuila, Mexico (the biggest automotive fabrication hubs in Mexico) going through Laredo, TX (where one of the busiest US Customs and Border Protection agencies is located), to Kansas City MO, US., one of the central hubs for manufacturing and assembly of vehicles in the US and another from Laredo, TX to Garyville, LA (where one of the biggest oil refineries is located).

## Results

**Fig 3:**
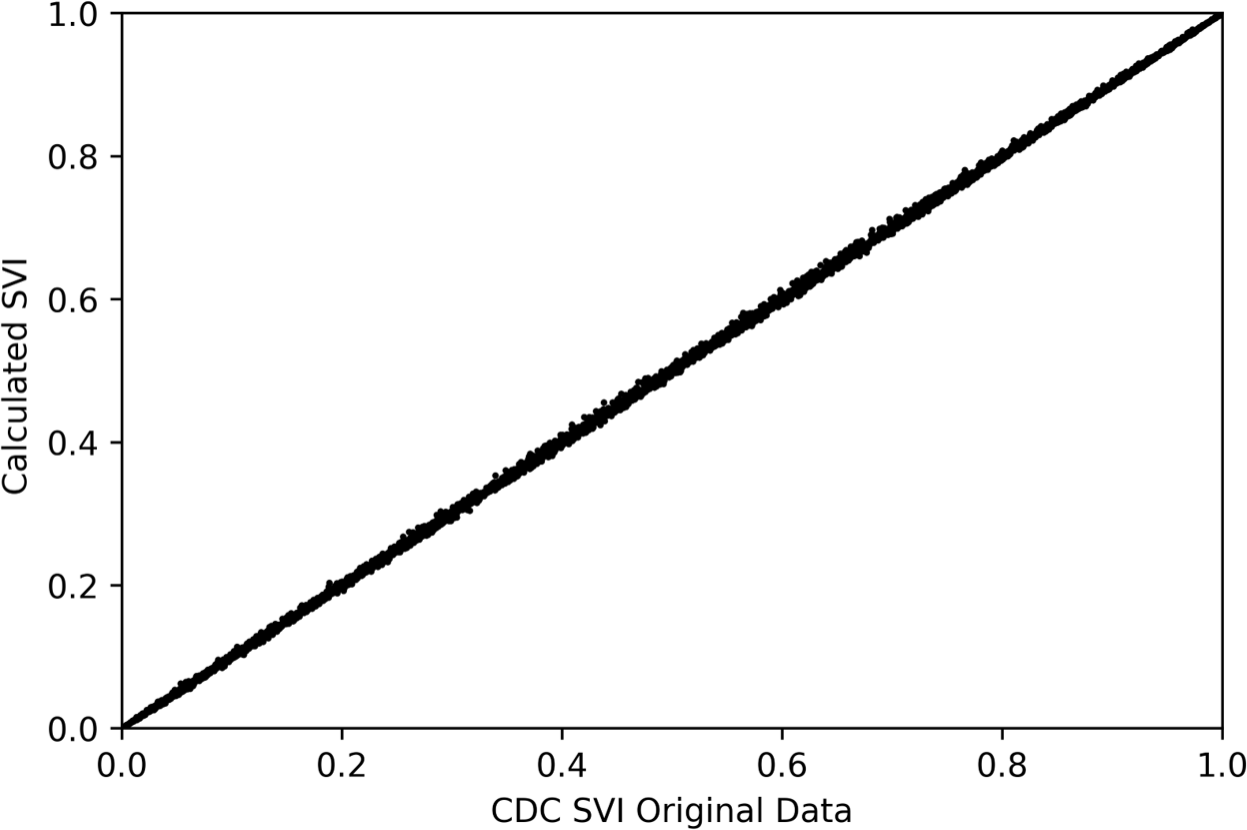
Comparison between the SVI CDC index and the SVI index based on the ECDF formulation. The 1-to-1 match shows that the reproducing the SVI index through ECDF produces the same estimates as the percentile ranking method used by the CDC’s SVI.

Fig 3 shows the comparison between estimates of the 2018 SVI and the authors’ estimates to reproduce it based on the ECDF approach (using the same data sources). The 2018 SVI for all counties across the country is compared against the reproduced SVI to show a one-to-one relationship. Reproduction of the index validates the ECDF approach to estimate the SVI.

**Fig 4:**
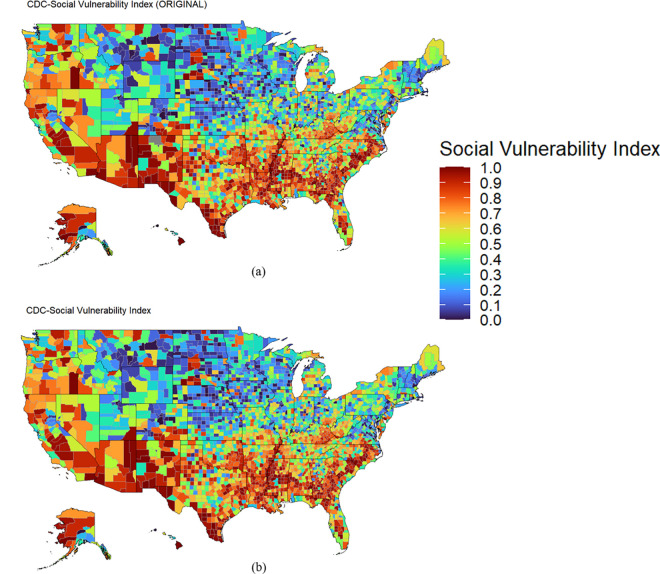
SVI spatial comparison between the CDC’s and the authors’ ECDF approach. The spatial distribution between the reproduced SVI index and the CDC published index is shown to be identical. a: CDC Social Vulnerability Index. b: Reproduced Vulnerability Index.

Fig 4 illustrates the similarity between the spatial distribution of SVI published by the CDC and the index formulated based on the ECDF approach. Figs 3 and 4 prove the equivalency between the CDC formulation and the authors’ SVI formulation using the ECDF approach.

**Fig 5:**
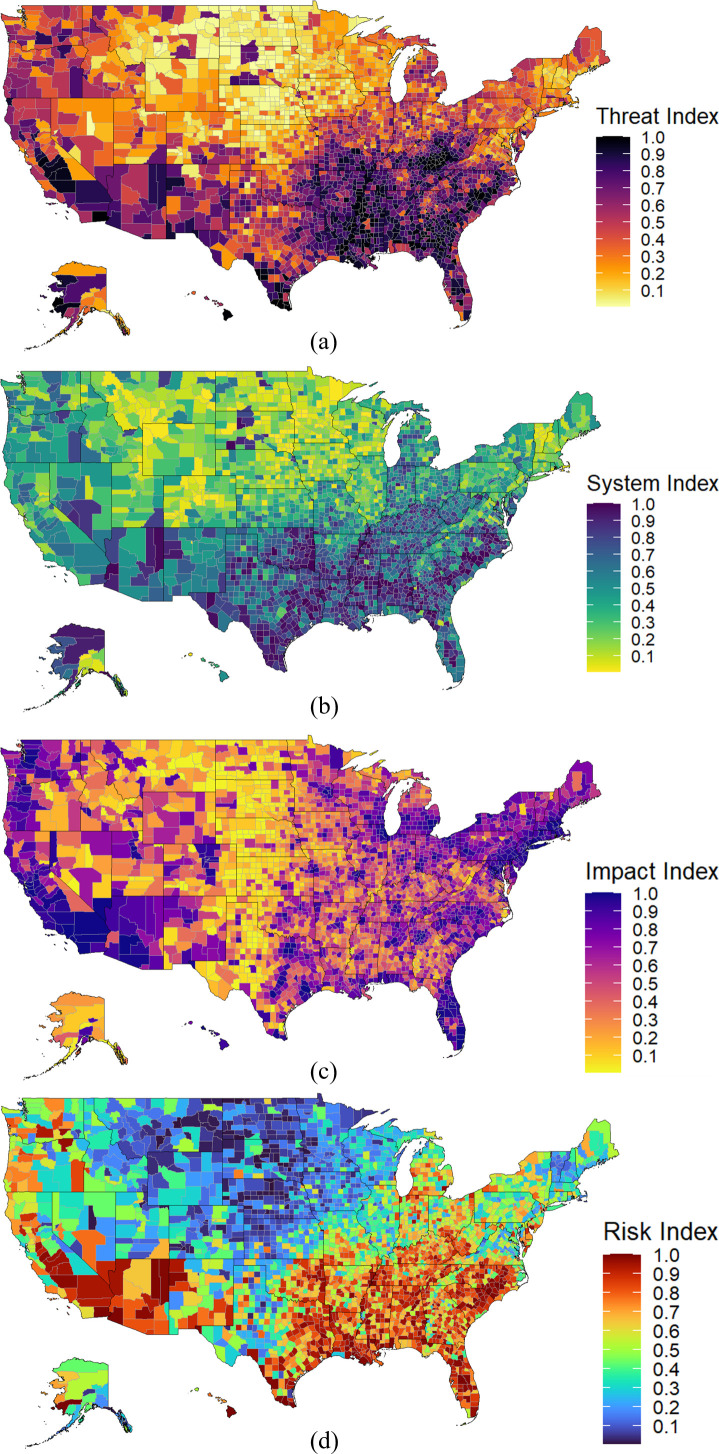
Pandemic social risk index map and its components. Each risk component index map corresponds to a representation of a subset of the metrics that were used to create risk index. a: Threat index map (6 variables). b: System index map (25 variables). c: Impact index map (1 variable). d: Risk index map.

Based on the variables listed in Table 1, it is possible to account for the influence of the regional health risk components associated to the COVID-19 pandemic. Using the SVI’s ECDF formulation, Fig 5 shows the pandemic social risk index (d) and its components, the aggregation of the Threats subtheme (a), the Vulnerable Systems subtheme (b), and the Metric of Impact subtheme (c).

**Fig 6:**
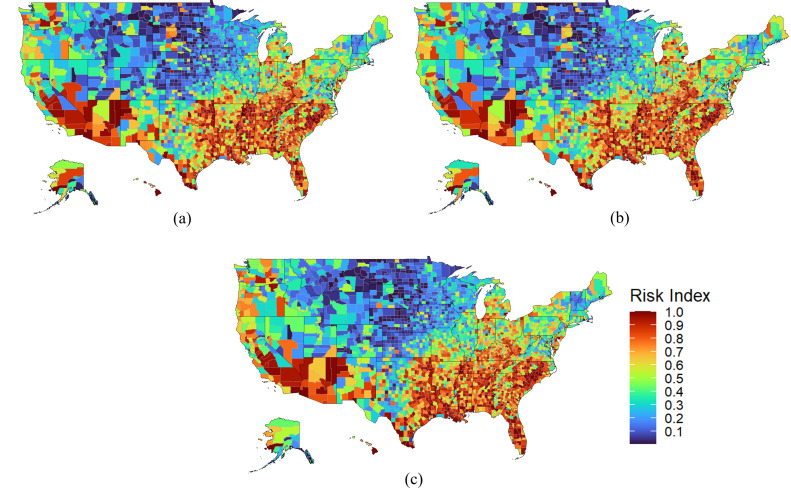
Temporal evolution of risk index. a: Early risk index map (December 17^th^ 2020). b: Mid risk index map (April 9^th^ 2021). c: Late risk index map (August 4^th^ 2021).

Fig 6 illustrates the temporal dynamics of the proposed risk index, showing the temporal evolution of the risk index on December 17^th^ 2020, April 9^th^ 2021, and August 4^th^ 2021, where it is observed minor regional changes and some significant local changes between them. This is because given that the Social Vulnerability Index (SVI) is inherently static (i.e., constructed from social and economic indicators derived from the U.S. Census Bureau’s 5-year estimates), only the metric representing the number of COVID-19 cases reported in the past seven days of the risk index ‘cut’ introduces temporal variability. As uniform weights were applied to all components of the risk index, its dynamic behavior is primarily driven by fluctuations in COVID-19 case counts. Consequently, the index does not account for critical temporal factors such as shifts in population distribution during the pandemic or changes in impacts stemmed from resource allocation since its temporal resolution is relatively much higher.. As mentioned before, this work reflects on an effort to formalize decision-making processes with a risk framework that account for the threats, vulnerability of participating systems, and metrics of impacts, and consequently shows its spatial and temporal limitations due to its conditioning of data temporal resolution. Notice that the earliest available dataset that CDC released regarding COVID-19 is December 17th of 2020 and no complete data from earlier date was made available [28].

**Fig 7:**
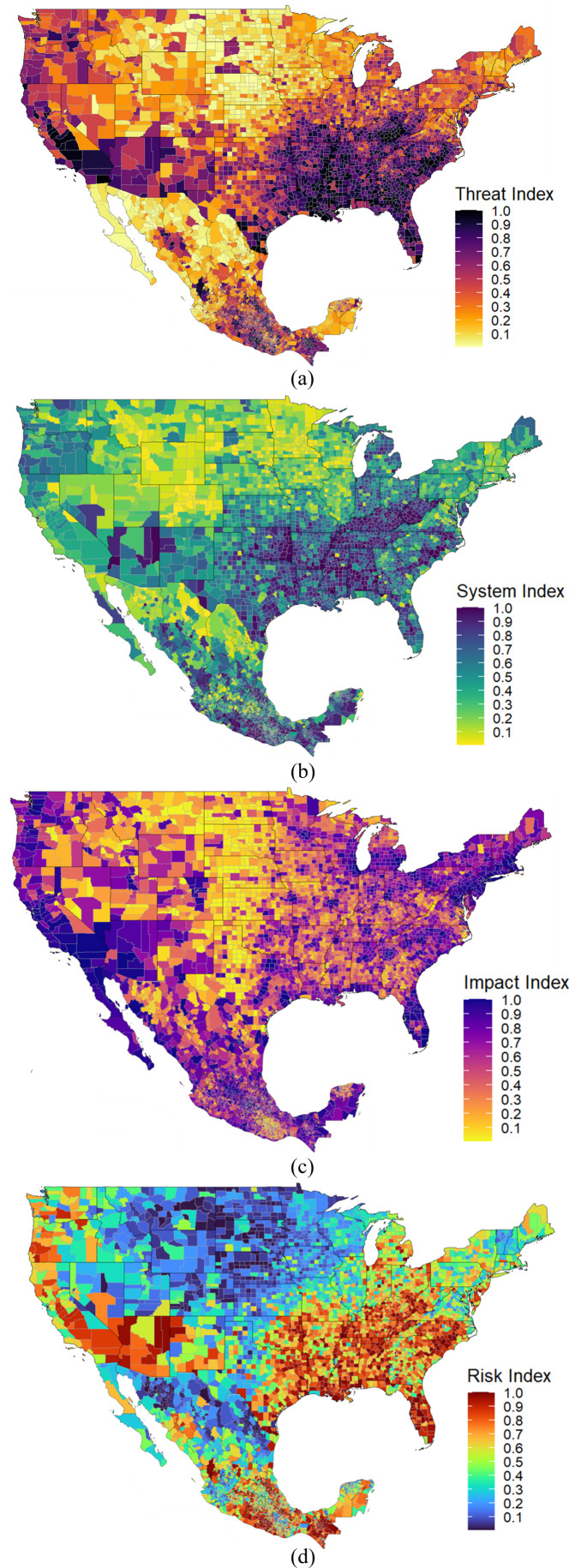
U.S.-Mexico pandemic social risk map. a: Threat index map. b: System index map. c: Impact index map. d: Pandemic social risk index map.

Fig 7 shows the bi-national US-Mexico maps corresponding to Threats, Vulnerable Systems, Metric of Impact, and the resulting map of the regional pandemic social risk index due to COVID-19 using the same index aggregation technique that was described to construct the U.S. risk index. A supply chain bi-national risk assessment between Mexico and United States has always been of strategic interest due to the volume of trade between the two countries. The regional state of risk of supply chains was exposed due to major disruption during the COVID-19 pandemic. The proposed pandemic social risk index (Fig 7) can serve as a reference to improve the understanding of past events to minimize future impacts on supply chains and other similar regional systems. It can help as well to outline strategic regional decision-making related to rear- and near- shoring.

**Fig 8:**
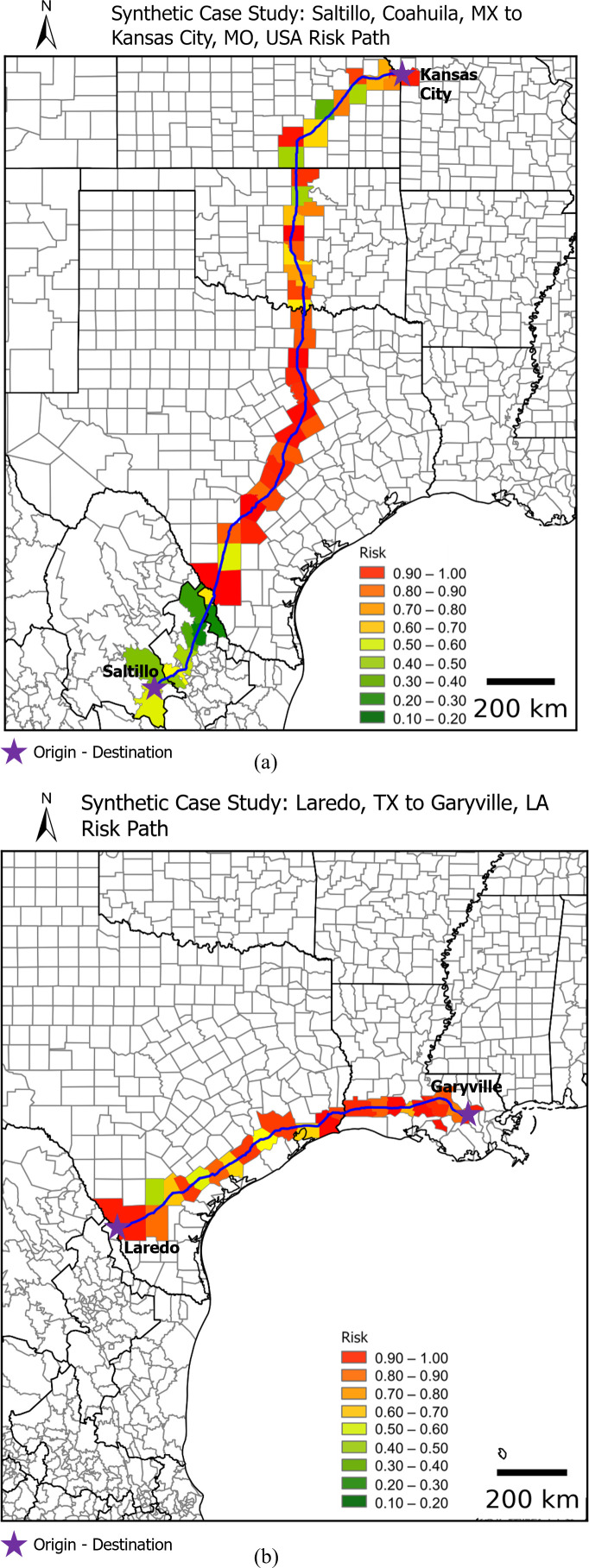
Example of a possible path connecting a: Saltillo, Coahuila (one of the biggest automotive fabrication hubs in Mexico) through Laredo, TX (one of the busiest ports of entry from Mexico) to Kansas City, MO (one of the central manufacturing hubs in the US).

To illustrate the applicability of integrating in a single map the U.S.-Mexico state of risk for a given set of threats, systems, and impact, two synthetic examples are presented in Fig 8 showing one possible route Saltillo, Coahuila, Mexico through a US custom agency in Laredo, TX, in the U.S. to Kansas City, MO, in the U.S. as well; and another possible route from Laredo, TX to Garyville, LA. The supply chain path-dependent risk is a weighted average of the traversed regions’ risk value relative to the distance travelled. The regional pandemic social risk index for both paths from Saltillo, Coahuila to Kansas City MO was estimated at 0.69, and the path from Laredo TX to Garyville LA was estimated at 0.80 respectiely, both based on the methodology presented in the previous section (Fig 2). The first example that which includes the crossing of the Mexico – USA border in Fig 8a was constructed using the U.S.-Mexico binational health risk map presented in Fig 7d and the second case study that shows a path within the U.S. only was constructed using the pandemic social risk index map presented in Fig 5d. Notice that the regional pandemic social risk index map due to COVID-19 is a useful metric in a path-based optimization procedures as it can be combined with other geographically-based metrics (e.g., distance) to find the most optimal path for supply chain transportation logistics.

## Discussion

This paper expands the concept of social vulnerability to risk. The selection of variables for the proposed pandemic social risk index was conducted to extend the scope of the CDC’s SVI by incorporating additional risk factors and expanding its applicability to account for impacts of the COVID-19 pandemic for regional supply chains between the U.S. and Mexico. Variables classified under Threats represented different dimensions of stressors addressing the *Hazard* risk component, while those classified under Vulnerable Systems represented the population’s socioeconomic and overall epidemiological characteristics addressing the *Vulnerability* risk component. And one variable was classified under Metrics of Impact to represent the affected population, addressing the *Consequences* risk component, which is not included in the original definition of the SVI. The spatial distribution of the proposed regional pandemic social risk index due to COVID-19 indicates a concentration of high social risk index on the eastern south part of the U.S. and also along the most populous border counties with Mexico. The Appalachian region of the U.S. also shows high risk index values according to the social stressors that have been included in the construction of the risk index. An interesting observation from the results presented in this manuscript is that Northern Mexico exhibits a relatively lower level of pandemic-related social risk, consistent with findings reported in other studies [33]. However, when compared with the dynamic response analysis by the same author, a significant difference is observed: Northern Mexico is no longer identified as the least vulnerable region, while Southern Mexico appears to exhibit reduced vulnerability.

The process of data collection presents several notable challenges. One primary difficulty lies in obtaining datasets that align temporally across different sources, as data corresponding to the same year are often unavailable. This limitation underscores the need for improved coordination and communication among data-collecting agencies, particularly given the interdependencies of public health metrics. Additionally, discrepancies in sampling strategies and estimation methodologies further complicate data harmonization (e.g., disease prevalence data, typically provided by the CDC, are estimated using sources such as the Behavioral Risk Factor Surveillance System (BRFSS) and U.S. Census data, each of which employs distinct approaches). Aggregating these metrics into a unified index inevitably compounds the individual uncertainties of each component—a challenge that cannot be fully addressed without full control over the initial data collection processes. Furthermore, integrating and harmonizing data from Mexico to construct a binational index introduces added complexity, stemming from differences in data collection protocols, population characteristics, and national definitions of vulnerable populations.

The pandemic social risk index, introduced above and based on a proposed ECDF methodology, can identify the concentration of counties and municipalities when mapped. The binational U.S.-Mexico version identifies regional risk (e.g., the center portion of USA and at the northern side of Mexico showing the lowest state of risk). However, one significant limitation of this method is that the risk index does not account for the population definition of each participating variable integrating the risk index, since the variable value aggregation assigns the same weight to each participating variable. An analysis of the temporal evolution of the pandemic risk index indicates a notable increase in risk levels in states such as Michigan and Oregon between December 17, 2020, and April 9, 2021. Conversely, a modest decline in the risk index is observed in certain border counties—particularly in Texas, the north-central United States, and Hawaii—during the subsequent period from April 9, 2021, to August 4, 2021.

Note that the simulated path shown in Fig 7 is one possible path but not necessarily the most optimal path since other considerations such as distance traveled, travel time, and fuel consumption would need to be considered in path optimization. These case studies serve as practical demonstrations of the proposed method for quantifying risk along a given path. Nonetheless, the proposed method to estimate a specific state of risk can be easily incorporated into other underlying systems such as supply chain analyses. Furthermore, factors can be considered as weight constraints in a standard path optimization problem.

## Conclusion

This paper demonstrates that the CDC’s Social Vulnerability Index (SVI) combines the effect of Threats and System Vulnerabilities when the SVI’s participating variables are classified according to a risk framework, defined in terms of three risk factors and their corresponding underlying events needed to reproduce decision-making: Threats → *Hazard*, Systems → *Vulnerability*, and Metrics of Impact → *Consequences*. An alternative approach to compute the SVI based on the Empirical Cumulative Distribution Function (ECDF) was proved to estimate the same SVI map as the one produced using the CDC ranking formulation, thus validating the ECDF approach. The ECDF approach allowed for the definition of the SVI’s variables in terms of population statistics, aligning it with probability theory and enabling to operate across states of information between variables. However, since the main focus on the formulation of a regional pandemic social risk index due to COVID-19 was based on the health system, and the aim to produce a path-dependent risk index for supply chains, it was favored a simple index formulation approach as SVI’s to produce preliminary regional estimates of risk. Additional variables were considered to include the influence of the COVID-19 as a Threat, account for health System vulnerabilities, and to incorporate a social Metric of Impact.

The resulting social risk index map and its corresponding risk component maps represent a simple and valuable resource to estimate the state of regional social risk. This can serve as a basis for overlaying other systems, such as the transportation networks of a supply from its origin to destination, allowing for the comparison of path-dependent state of risks. Two synthetic examples were formulated to demonstrate the estimation of a path dependent regional health risk due COVID-19 for two possible routes, from Saltillo, Coahuila, Mexico through Laredo, TX, USA to Kansas City, MO, USA, and another from Laredo TX to Garyville LA, highlighting the practical applicability of the proposed regional pandemic social risk index due COVID-19 in supply chain risk assessment. The index can be combined with other geographically based metrics, such as trajectories and energy consumption, as part of a more complex optimal optimization system during major disruption events.

The proposed approach provides a foundation for decision-makers to assess and manage risks associated with pandemics such as COVID-19, particularly in the context of supply chain disruptions between the U.S. and Mexico. By integrating this risk assessment methodology into supply chain management practices, stakeholders can develop more resilient strategies to mitigate the impacts of future pandemics on regional and global trade. Further research is ongoing to address the statistical limitations of the SVI aggregation approach when formulating spatial-temporal states of risk. A separate formulation effort in progress will follow to this work, aiming to account for the dynamic nature of risk assessment and decision-making in the face of a pandemic like COVID-19, or other major disruptive events (i.e., geopolitics, climate change, immigration, etc.), which will enhance the robustness and applicability of the proposed risk index

## Supporting information

S1 Text Description and construction of Empirical Cumulative Density Function. (DOCX)
