# Supplementary material for: A pandemic risk index to improve supply chains decision-making between US and Mexico: A COVID-19 case study
Source: PLoS One. 2025 Sep 11;20(9):e0327526. doi: 10.1371/journal.pone.0327526 (PMC12425231; doi:10.1371/journal.pone.0327526)
Supplement: S1 Text — (DOCX) [file pone.0327526.s001.docx]

**S1 Text. Description and construction of Empirical Cumulative Density Function.**

The Empirical Cumulative Density Function (ECDF) is constructed from a single variable (i.e., dataset) and is a non-parametric estimator of the Cumulative Density Function (CDF). It provides an estimate of the proportion of data points that are less than or equal to a particular value. The steps to construct an ECDF is as following,

1. Sort the data in ascending order, $x_{1}, x_{2}, x_{3},\ldots, x_{n}$.
2. Assign ranks to the sorted data points from 1 to $n$.
3. For each data point $x_{i}$, the ECDF value is calculated using

|  | $F_{n}\left( x_{i} \right)=\frac{i}{n}$ | (2) |
| --- | --- | --- |

The ECDF is then plotted as a step function where the x-axis represents the sorted data values $x_{i}$ and the y-axis represents the corresponding ECDF values $F_{n}(x_{i})$.
